# Genome-wide methylation profiling identify hypermethylated HOXL subclass genes as potential markers for esophageal squamous cell carcinoma detection

**DOI:** 10.1186/s12920-022-01401-x

**Published:** 2022-11-29

**Authors:** Qiuning Yu, Namei Xia, Yanteng Zhao, Huifang Jin, Renyin Chen, Fanglei Ye, Liyinghui Chen, Ying Xie, Kangkang Wan, Jun Zhou, Dihan Zhou, Xianping Lv

**Affiliations:** 1 Otorhinolaryngology Hospital, The First Affiliated Hospital of Zhengzhou University, Zhengzhou, 450052 China; 2 Department of Transfusion, The First Affiliated Hospital of Zhengzhou University, Zhengzhou, 450052 China; 3 Department of Pathology, The First Affiliated Hospital of Zhengzhou University, Zhengzhou, 450052 China; 4 Wuhan Ammunition Life-tech Company, Ltd., Wuhan, Hubei China

**Keywords:** DNA methylation, Esophageal squamous cell carcinoma, HOXL subclass, Early detection markers

## Abstract

**Background:**

Numerous studies have revealed aberrant DNA methylation in esophageal squamous cell carcinoma (ESCC). However, they often focused on the partial genome, which resulted in an inadequate understanding of the shaped methylation features and the lack of available methylation markers for this disease.

**Methods:**

The current study investigated the methylation profiles between ESCC and paired normal samples using whole-genome bisulfite sequencing (WGBS) data and obtained a group of differentially methylated CpGs (DMC), differentially methylated regions (DMR), and differentially methylated genes (DMG). The DMGs were then verified in independent datasets and Sanger sequencing in our custom samples. Finally, we attempted to evaluate the performance of these genes as methylation markers for the classification of ESCC.

**Results:**

We obtained 438,558 DMCs, 15,462 DMRs, and 1568 DMGs. The four significantly enriched gene families of DMGs were CD molecules, NKL subclass, HOXL subclass, and Zinc finger C2H2-type. The HOXL subclass homeobox genes were observed extensively hypermethylated in ESCC. The HOXL-score estimated by *HOXC10* and *HOXD1* methylation, whose methylation status were then confirmed by sanger sequencing in our custom ESCC samples, showed good ability in discriminating ESCC from normal samples.

**Conclusions:**

We observed widespread hypomethylation events in ESCC, and the hypermethylated HOXL subclass homeobox genes presented promising applications for the early detection of esophageal squamous cell carcinoma.

**Supplementary Information:**

The online version contains supplementary material available at 10.1186/s12920-022-01401-x.

## Background

Esophageal cancer (EC) is one of the top 10 fetal malignant tumors worldwide, with a five-year overall survival rate of less than 20% [1]. The incidence of EC in men is four to five times higher than in women, and it predominantly occurs in East Asia as well as Eastern and Southern Africa [2]. Esophageal adenocarcinoma (ESCA) and esophageal squamous carcinoma (ESCC) are two major histologic subtypes of EC, with ESCC being the most common type [3]. Risk factors for developing EC are complex and vary among different histologic subtypes. ESCA is prevalent in Caucasian populations, and risk factors include obesity, gastroesophageal reflux disease, and Barrett's esophagus. In contrast, ESCC is the predominant type of EC in East Asia and sub-Saharan Africa [4], while its main risk factors are papillomavirus infection, smoking, alcohol consumption, and hot foods [5]. Currently, EC is the fourth leading cause of cancer-related deaths in China, and ESCC is the most frequently diagnosed type (accounting for more than 90% of all EC cases), which is different from that in Western countries [6]. The morbidity and mortality of ESCC in China increase with age. The disease risk rapidly escalates after age 40, and the mortality rises after age 50 in a population [7].

Various staging strategies have been proposed to guide the clinical management of esophageal cancer better, and the TNM staging criteria (8th edition) jointly developed by the American Joint Commission on Cancer and the Union for International Cancer Control in 2017 is one of the widely used references [8]. In addition, NCCN has also released the clinical practice guidelines for esophageal cancer [9]. Accurately staging ESCC is critical for the clinical management of this disease. According to the eighth edition TNM categories, patients with lesions < 2 cm, tumors limited in the mucosal lamina propria or muscularis mucosae, moderate to high differentiation, and low risk of lymph node metastasis, local recurrence, or distant metastasis are classified as early stage. The high mortality and low 5-year survival rate of ESCC are mainly attributed to its advanced stage at diagnosis. The 5-year survival rate of early-stage ESCC improved from less than 20 to 80–90% after surgical or endoscopic resection [10]. Therefore, early detection can help reduce the incidence of ESCC and prolongs patient prognostic survival time. Traditionally, endoscopy has been the first choice for ESCC screening and can detect intraepithelial neoplasia, such as dysplasia and local non-invasive carcinoma, in asymptomatic patients, which is recognized as the major precancerous lesions [11]. A long-term follow-up showed that appropriate treatment of esophageal squamous epithelial dysplasia and early-stage ESCC after endoscopic screening reduced the disease mortality in the average-risk population in China [12]. In most developing countries, however, extensive endoscopic screening is not feasible, given the cost-effectiveness. In contrast, inexpensive non-endoscopic esophageal sampling methods were proposed for ESCC screening. These sampling techniques combined with cytological examination have displayed some advantages, although the results of several studies are not satisfactory [13]. The potential utility of molecular diagnostic markers has been demonstrated for early detection of EC, including cell-free miRNAs and genomic abnormally methylated DNA [14, 15]. Although the combination of DNA methylation and esophageal sampling techniques has presented a high accuracy in discriminating ESCC from normal controls [16, 17], available methylation markers are still inadequate, and minimally invasive detection techniques based on blood methylation markers are urgently needed to be developed.

Several abnormal methylated genes on ESCC have been reported so far, and they are grouped into DNA damage repair, cell cycle regulation, cell adhesion, proliferation, and other biological categories [18]. These identified hypermethylated genes include *MGMT*, *MLH1* (DNA damage repair), *CDKN2A*, *CHFR* and *CDKN2B* (cell cycle regulation), *APC* and *SOX17* (Wnt signaling pathway), *RUNX3* and *DACH1* (transforming growth factor ⁃β), and *CDH1*, *TFF1*, *TFPI2* (other biological functions). Previous studies suggested that some hypermethylated genes occurred in early-stage ESCC, including the well-known genes *MGMT* [19], *CDKN2A* [20], *MLH1* [21], and *CDH1* [22], while some are hypermethylated in late-stage such as *CHFR* [23], and others, such as hypermethylated *APC* are not associated with ESCC stage [24]. Although these genes are reported to be significantly hypermethylated on ESCC, their hypermethylation frequencies are not satisfied (from 30 to 60%) [18], and their potential for ESCC detection is rarely investigated.

The widespread use of high-throughput techniques in ESCC allowed us to view the landscape of genomic features of this disease. DNA methylation, as one type of epigenetic modification, has received the most attention. Recent findings suggested that aberrant DNA methylation can be used as a signal for the early detection of ESCC. For example, hypermethylated *CDKN2A*, *CDKN2B,* and *TFF1* were found in the early stages of ESCC [25]. In a study combining 850 k and 450 k methylation data in ESCC, DNA methylation was more frequent and robust in tumor tissues than normal mucosa, with 1/4 of the hypermethylated genes (165 genes) being observed in early ESCC (stage I-II) [26]. Investigations based on whole-genome bisulfite sequencing (WGBS) data revealed that about 2% (~ 36,000) of CpG sites were in hypermethylated status, including inactivated negative regulators of the Wnt pathway due to aberrant methylation [27]. These pioneering studies provide a treasure trove to identify more effective biomarkers for the early detection of ESCC.

Although many studies have revealed aberrant DNA methylation in ESCC, most have focused on only a tiny fraction of the genome. The widely used 450 k methylation microarray data cover approximately 480,000 CpG sites [28], representing the local genomic methylation status. Therefore, it is necessary to explore the methylation features from a global perspective. In this study, we investigated the methylation patterns of ESCC in genome-wide using WGBS data and elaborated the possible biological functions of these aberrantly methylated genes. Finally, the ability to detect ESCC was shown by integrating multiple datasets and screening several methylation genes as potential markers.

## Methods

### Data preparation and preprocessing

GSE149608 [27] and GSE52826 [29] datasets were retrieved from the GEO database (https://www.ncbi.nlm.nih.gov/geo/) as both consisted of tumor and paired normal samples. The two datasets are generated by whole-genome bisulfite sequencing (WGBS) and Illumina HumanMethylation450k, covering over 18 million and 480,000 CpG sites. The methylation value of each CpG site is represented by the percentage of methylated reads in total reads that cover this site for WGBS data and by the signal intensity value of β (range 0–1) for 450 k microarray data. TCGAbiolinks tools [30] are used to download the level 3 methylation data, the corresponding clinical features of ESCC, and paired normal samples from The Cancer Genome Atlas (TCGA) Program (https://portal.gdc.cancer.gov/). CpG sites are removed during the data preprocessing if the methylation values were 0 in all samples of WGBS dataset or the β values are missing in more than 90% of the samples in 450 k dataset. Then, KNN algorithm is used to fill the missing values.

The preprocessed datasets are shown in Table 1, containing 29 normal samples and 109 tumor samples. The clinical information of GSE149608 is collected from GEO database simultaneously but is not consistent with the reference paper. Here, we determined their exact clinical characteristics (Additional file 1: Table S1) according to the information provided by the reference, which includes two early-stage patients (patient3 and patient10), five intermediate-stage patients (patient 2, patient 5–8), and two late-stage patients (patient1 and patient4) stages. All cases in GSE52826 dataset are early-stage ESCC (Additional file 1: Table S2).

**Table 1:** The three eligible datasets used in this study

Dataset	Normal (n)	Tumor (n)	Platform
GSE149608	9	9	WGBS
GSE52826	4	4	Illumina HumanMethylation450k
TCGA ESCC	16	96	Illumina HumanMethylation450k

### Human tissue and blood samples

Twenty formalin-fixed paraffin-embedded esophageal squamous carcinoma and adjacent normal sample were collected from the department of pathology of the First Affiliated Hospital of Zhengzhou University in the study. Meanwhile, twenty healthy blood samples were obtained from the blood transfusion department, where the residual blood samples were collected from healthy individuals after blood donation. The healthy blood samples are included because they represent the methylation status of candidate targets on healthy individuals and will also facilitate investigating their potential role as blood diagnostic markers. Twenty plasma samples were also collected from ESCC patients, but only 13 samples obtained adequate cell-free DNA amounts. Clinical features of the samples are displayed in Additional file 1: Table S3. Referring to the NCCN guidelines, in this study, we define Tis (high-grade dysplasia), T1 (T1a and T1b), and some T2 (without lymph node metastasis and distal metastasis) as early-stage ESCC. All individual identifiers have been removed. The Ethics Committee of the First Affiliated Hospital of Zhengzhou University approved this study (approval number 2020-KY-0152).

### Identification of differentially methylated genes

Since the vast majority of CpG sites in the whole genome are covered in WGBS data, representing the comprehensive epigenetic information, the GSE149608 dataset was selected to identify differentially methylated CpG sites (DMC) between tumor and paired normal samples using paired student t-test, with P-value < 0.05 and fold change ≥ 2 as the significance threshold. We defined hypermethylated DMCs in normal and tumor samples as NC-DMCs and ESCC-DMCs respectively. The methylation status of adjacent CpG sites are usually highly coupled with each other, and they tend to locate in a small genomic region [31], therefore we further identified the differentially methylated regions (DMR) based on DMCs using a modified sliding window approach described in our previous study [32]. Briefly, to facilitate developing methylation specific PCR (MSP) assay, the maximum length of DMRs is set to 100 bp, and each DMR contains at least three DMCs, and the distance between two adjacent DMCs is less than 50 bp. NC-DMRs and ESCC-DMRs were then identified separately, and the genes covered by NC- or ESCC-DMRs were defined as differentially methylated genes (NC-DMG or ESCC-DMG), except for those overlapped with both NC- and ESCC-DMRs. Since ESCC-DMRs are more eligible for MSP, only ESCC-DMGs are selected for subsequent analysis.

### Function enrichment analysis

Gene ontology (GO) and KEGG [33] pathway enrichment analysis were performed using the ‘clusterProfiler’ R package for NC-DMGs and ESCC-DMGs. All human genes are used as the background with q-value < 0.05 as the significance threshold. Gene family enrichment analysis was implemented by integrating gene family terms provided by HGNC (HUGO Gene Nomenclature Committee) [34] and the chi-square test. Briefly, the human gene family data are downloaded from HGNC (www.genenames.org), which records the human genes and their corresponding families. For each family, we counted the number of DMGs belonging to this family. The overrepresented gene families are assessed with all human genes and DMGs as background. The chi-square test *P-value* < 0.05 is selected as the significance threshold to determine enriched families for NC-DMGs and ESCC-DMGs.

### DNA extraction and BS-treatment

DNA of tissue and blood samples are extracted using paraffin-embedded tissue DNA Rapid Extraction Kit (TIANGEN®, Beijing) and TIANamp Genomic DNA Kit (TIANGEN®, Beijing), respectively. BS-treatment and purification for the extracted DNA were subsequently carried out using the Nucleic Acid Purification Kit (Ammunition®, Wuhan). The basic principle of BS-treatment is that unmethylated cytosines in denatured DNA can be converted to uracil by bisulfite ions, while methylated cytosines are remained. The methylation status then can be determined by methylation-specific PCR.

### Methylation-specific PCR and sanger sequencing

Methylation-specific PCR experiments and Sanger sequencing were performed to determine the methylation status of candidate targets. Two genes, *HOXD1* and*HOXC10*, are selected for MSP because they show the best abilities to discriminate cancer samples from normal samples. Besides, high densities of differentially methylated CpG sites were are found within the two genes, which allowed us to design appropriate MSP primers. The designed primers for *HOXD1* and *HOXC10* are listed in Table 2. The amplified regions cover DMR4, DMR5, and DMR6 of *HOXD1*, and DMR1 and DMR2 of *HOXC10* (Additional file 1: Table S4). The fully unmethylated and methylated DNA fragments for the two targets were synthesized as negative and positive controls. The MSP amplification system is 20ul, including 7ul of dd-water, 10ul of 2 × T5 Fast qPCR Mix (SYBR Green I), 0.5ul of forward and reverse primers (10 uM), and 2ul of BS-converted DNA. PCR reaction is pre-denaturation at 95 °C for 1 min, denaturation at 95 °C for 10 s, annealing and extension at 60 °C for 45 s. The melt curve reaction is 95 °C ~ 15 s, 60 °C ~ 1 min, and 95 °C ~ 15 s. Quantitative real-time PCR is performed on a 7500 device (Thermo Fisher, USA). The MSP products were then used for Sanger sequencing. After BS-treatment, the methylated CpG site would remain C, and the un-methylated C would be T. Therefore, the methylation status of target CpG sites can be determined according to the result of Sanger sequencing.

**Table 2:** Primers used for methylation-specific PCR

Gene	Primer	Sequence (5′ –> 3′)	Position	Length (bp)	CpG sites
*HOXD1*	Forward	CCCCGTTGTAGGTAAATTCGTC	Chr2:177054528–177054672	144	10
	Reverse	GGGACTATCTCGATACGCCGA			
*HOXC10*	Forward	TATTTGACGCGAGAGCGTCG	Chr12: 54383074–54383217	147	7
	Reverse	TTAAAATTAAAAATCAATTCCCG			

### ROC analysis

For the candidate target genes, we view the sample types (normal or tumor) as the response variable of their methylation values to develop a classifier for ESCC using logistic regression. The logistic probability, herein defined as risk scores for samples, was then estimated. Typically, samples will be recognized as positive if the risk scores are > 0.5 and vice versa as negative. This study used ROC curve to determine the appropriate threshold and AUC value to assess the classifier performance. The optimal threshold was locked when Youden index reached the maximum. Samples are subsequently divided into a positive group if the scores are higher than this threshold or a negative group if the scores are less than this threshold. All samples were classified into four categories, true positive (TP), true negative (TN), false positive (FP), and false negative (FN) according to their sample types and predicted types. The sensitivity and specificity are calculated using the following formulas:

$$\begin{aligned} Sensitivity & = \frac{TP}{{TP + FN}} \\ Specificity & = \frac{TN}{{TN + FP}} \\ \end{aligned}$$

### Statistical analysis

Data preprocessing, statistical analysis, and other analysis in this study are implemented in R software (v3.6.1). For continuous variables, paired student t-test is used for comparisons of paired normal and tumor samples, and the wilcoxon rank-sum test for unpaired datasets. The Kruskal–Wallis test is performed for comparisons of multiple groups. For category variables, chi-square test is used to estimate the difference between groups. Logistic regression is implemented using the R package ‘glmnet’ with the parameter ‘family = binomial’. ROC analysis is conducted using the package ‘pROC’ [35] with default parameters.

## Results

### Landscape of the aberrantly methylated CpGs between normal and ESCC

**Fig. 1:**
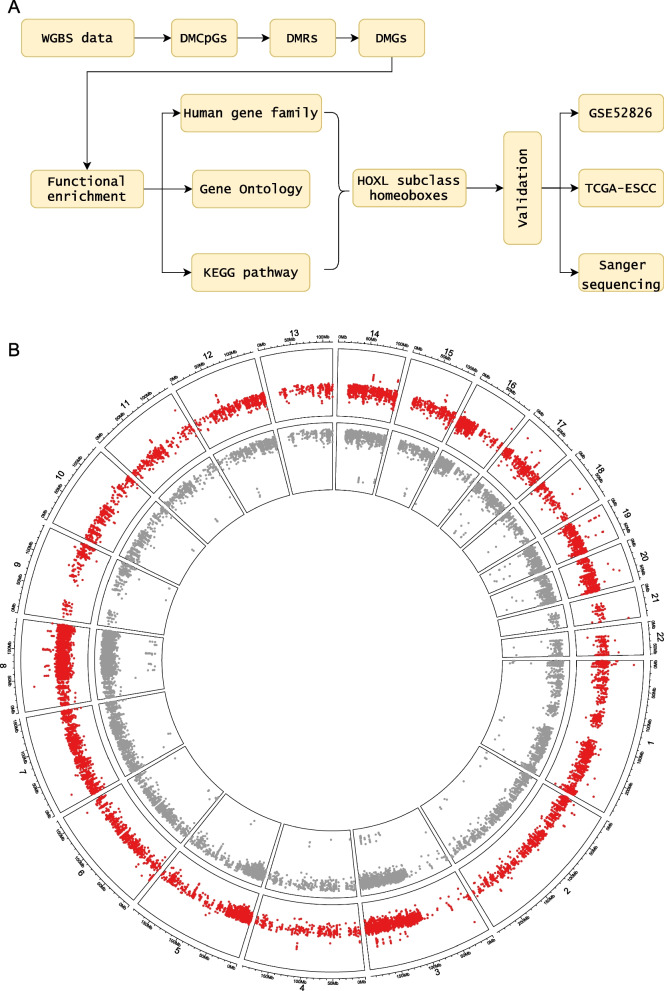
Methylation profiling of ESCC and paired normal samples. **A** An overview of this study. TCGA pan-cancer included the 9 most common cancer types: esophageal carcinoma (ESCA), esophageal squamous cell carcinoma (ESCC), stomach adenocarcinoma (STAD), colorectal cancer (CRC), liver hepatocellular carcinoma (LIHC), pancreatic adenocarcinoma (PAAD), breast invasive carcinoma (BRCA), lung adenocarcinoma (LUAD), lung squamous cell carcinoma (LUSC). **B** The identified DMCs across the whole genome. The red and gray points represented the ESCC-DMCs and NC-DMCs respectively. Chromosome X and Y were excluded in **B**. *WGBS* whole-genome bisulfite sequencing, *DMC* differentially methylated CpG, *DMR* differentially methylated region, *DMG* differentially methylated gene

The flowchart of this study is presented in Fig. 1A. The following analyses were performed to survey the landscape of ESCC methylome. Firstly, differentially methylated CpG sites between normal and tumor samples were identified using WGBS data. Due to the highly coordinated CpG sites being often tightly coupled with each other, we further identified differentially methylated regions (DMR) by a modified sliding window method. Differentially methylated genes (DMG) were then identified based on the genomic coordinates of DMRs and genes (see the method). Here we define DMR or DMG as NC-DMR and NC-DMG if their methylation levels are higher on normal controls than tumor samples and vice versa as ESCC-DMR and ESCC-DMG. GO and KEGG pathway enrichment analyses were conducted to explore the potential functions of these DMGs. We also performed gene family analysis to identify significantly enriched gene families. The HOXL subclass homeobox family was selected to validate on two independent datasets and by Sanger sequencing in our custom samples. We further investigated the potential utility of the family genes as markers for cancer detection.

We identified 438,558 DMCs, including 361,341 NC-DMCs and 69,278 ESCC-DMCs (Fig. 1B; Additional file 1: Table S5). Chromosome 8 has the most NC-DMCs, while the most ESCC-DMCs were in chromosomes 1 and 2 (Additional file 1: Fig. S1A). The distance between ESCC-DMCs is smaller than that of NC-DMCs (Additional file 1: Fig. S1B). Further investigations indicated that the methylation levels between adjacent DMCs are strongly correlated, and the correlation coefficients of adjacent ESCC-DMCs are much higher than NC-DMCs (0.73 vs. 0.70, *P* < 0.05, Additional file 1: Fig. S1C).

### Identification of DMGs

**Fig. 2:**
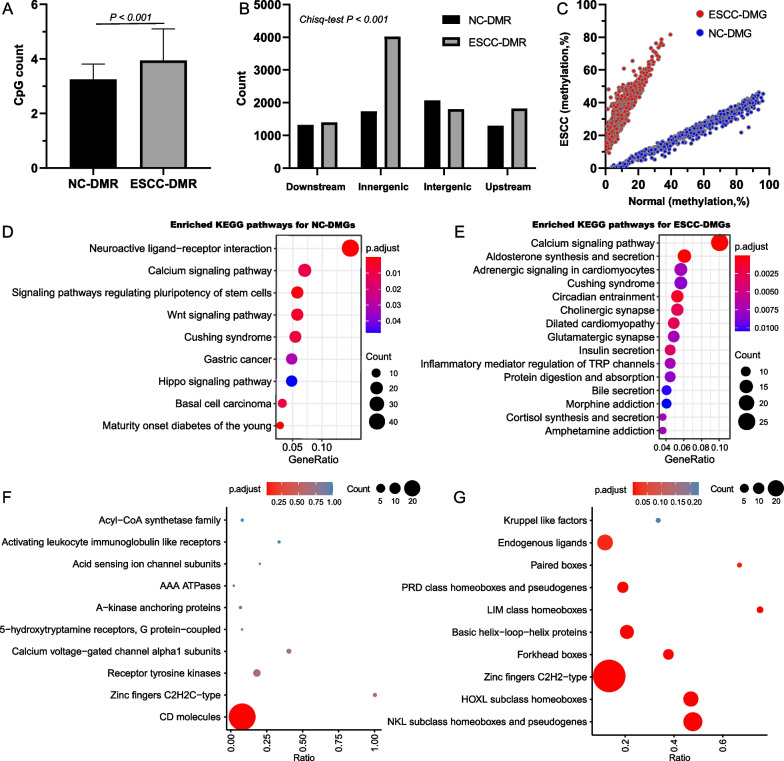
Functional enrichment analysis of differentially methylated genes. **A** The number of DMCs in ESCC-DMRs and NC-DMRs. **B** The genome distribution of ESCC-DMRs and NC-DMRs. **C** The methylation levels of DMGs in normal and ESCC samples. **D** and **E** The enriched KEGG pathways of NC-DMGs (**D**) and ESCC-DMGs (**E**). **F** and **G** The top 10 enriched gene families of NC-DMGs (**F**) and ESCC-DMGs (**G**). The circle size in **D**–**G** represented the mapped gene numbers, and color indicated the enriched p value

We obtained 6422 NC-DMRs and 9040 ESCC-DMRs based on the DMCs. Similarly, most NC-DMRs are found in chromosome 8, while the majority of ESCC-DMRs are in chromosome 1 and 2 (Additional file 1: Fig. S2A). A smaller distance is also observed for adjacent ESCC-DMRs than that of NC-DMRs (Additional file 1: Fig. S2B). The average DMC count of ESCC-DMRs is higher than that of NC-DMRs (Fig. 2A), and ESCC-DMRs tend to be located in inner-genic regions, whereas NC-DMRs more often located in intergenic regions (Fig. 2B). According to the genomic coordinates of DMRs and genes, we identified 733 NC-DMGs and 906 ESCC-DMGs, of which 71 genes are shared by both (Additional file 1: Table S6). Chromosome 19 has the largest proportion (9.57%), but not significantly higher than the other chromosomes such as chromosome 1 (9.10%). The methylation values are able to separate ESCC-DMGs and NC-DMGs clearly, and ESCC-DMGs show more concentrated than NC-DMGs (Fig. 2C, 1st–3rd quantile: [29.32–43.64] vs. [13.86–34.14]). Functional enrichment analysis reveals that neuroactive ligand-receptor interaction is the most significantly enriched pathway for NC-DMGs (Fig. 2D). For ESCC-DMGs, calcium signaling pathway is the most significantly enriched pathway (Fig. 2E). The term of CD molecules is the major enriched gene family for NC-DMGs, whereas NKL subclass (Fig. 2F; Additional file 1: Table S7), HOXL subclass, and Zinc fingers C2H2-type are the 3 top-ranked enriched families for ESCC-DMGs (Fig. 2G; Additional file 1: Table S8).

### The aberrant methylation of HOXL subclass homeoboxes

**Fig. 3:**
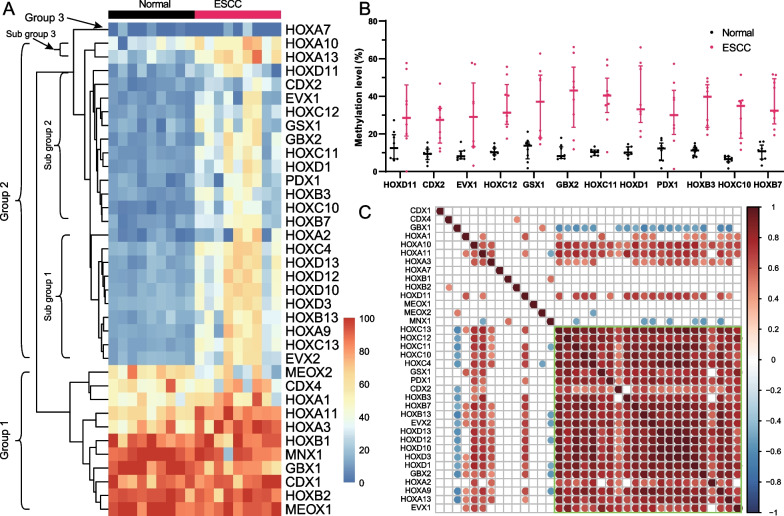
Methylation patterns of HOXL subclass homeoboxes. **A** The methylation levels of HOXL subclass homeoboxes genes in normal and ESCC samples. **B** The methylation levels of subgroup 2 genes between normal and ESCC samples. **C** The methylation correlation of HOXL subclass homeobox genes. The correlation coefficients were estimated using Pearson’s method. The β values of each gene in **A**–**C** were calculated using TCGA ESCC dataset

We selected the second-ranked HOXL subclass homeobox family for further analysis, as more than half (n = 24, 66.67%) of the genes were identified as ESCC-DMGs, which is the highest than the other two families (32/57 = 56.14% for NKL subclass and 70/648 = 10.8% for Zinc fingers C2H2-type). K-means clustering shows that the 36 genes can be clustered into 3 groups (Fig. 3A). Group 1 consists of 11 genes that are hypermethylated on both normal and ESCC samples. Twenty-four genes, all of which are ESCC-DMGs, are in group 2. Furthermore, group 2 genes are divided into three sub-groups, subgroup 1 (n = 10), subgroup 2 (n = 12), and subgroup 3 (n = 2). Only one gene, *HOXA7*, is in group 3 and shows low methylation levels on normal and ESCC samples. Overall, genes of subgroup 2 show the lowest methylation levels on normal samples than sub group 1 and sub group 3 (Fig. 3B). Correlation analysis reveals significant positive correlations between group 2 genes, except for *MNX1* and *GBX1* (Fig. 3C).

### Validation of sub-group 2 genes of HOXL subclass homeobox

**Fig. 4:**
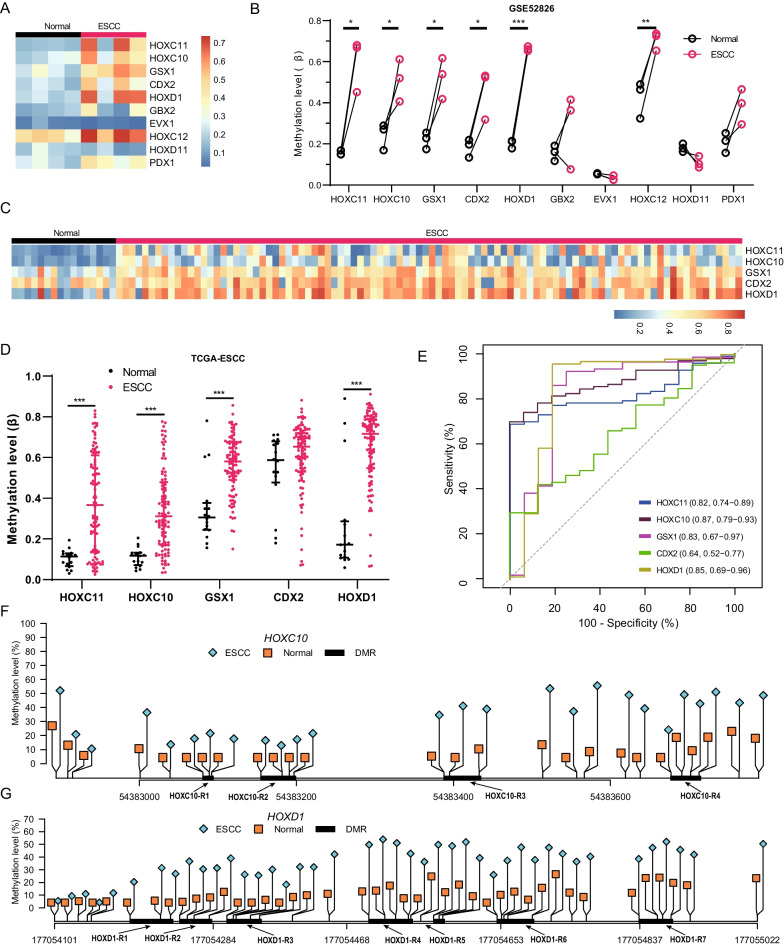
Methylation patterns of sub-group 2 genes in validation sets. **A** The methylation levels of sub-group 2 genes in normal and ESCC samples in GSE52826 dataset. **B** The differentially methylated six genes between ESCC and paired normal samples. Paired student t-test was used to estimate significant p values. **C** The methylation levels of five genes in normal and ESCC samples in TCGA-ESCC. **D** The differentially methylated four genes between ESCC and normal samples. The Wilcoxon rank sum test was used to estimate significant p values. **E** ROC curves of the four genes in classifying TCGA-ESCC and normal samples. **F**–**G** The identified ESCC-DMRs of *HOXC10* and *HOXD1* represented by R1–R4 and R1-R7 respectively

Using GSE52826 dataset, we verified the methylation characteristics of subgroup 2 genes (n = 10) in ESCC and paired normal samples (Fig. 4A). After the exclusion of outlier samples (GSM1276746 and matched GSM1276750) (Additional file 1: Fig. S3), six genes are validated hypermethylated in tumor samples (Fig. 4B). Since *HOXC12* shows high methylation levels in normal samples, we further investigated methylation patterns of the rest five genes on TCGA ESCC dataset (Fig. 4C). The methylation of *HOXC11*, *HOXC10,* and *HOXD1* in normal samples are lower than *GSX1* and *CDX2* (Fig. 4D), suggesting their low methylation background. ROC analysis indicated that methylation values of *HOXC10* and *HOXD1* show the best performance in discriminating ESCC from normal samples, with both AUC reached 0.85 (Fig. 4E). Further investigations revealed that four DMRs located in *HOXC10*, and HOXC10-R3 presented the largest delta methylation value (Fig. 4F). *HOXD1* contains seven DMRs, with HOXD1-R4 shows the largest delta methylation value (Fig. 4G). When stratified TCGA ESCCs by different pathological stages, both genes show hypermethylated across all stages, including stages IA and IB (Additional file 1: Fig. S4).

### The performance of HOXL-score for ESCC classification

**Fig. 5:**
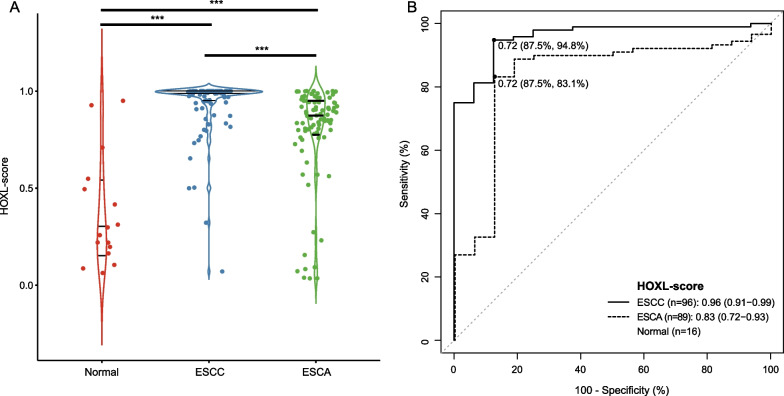
The performance of HOXL score for ESCC classification. **A** The HOXL scores of normal, ESCC and ESCA samples. For two group comparisons, the Wilcoxon rank sum test was used. For three group comparison, the Kruskal test was used. The three lines from top to bottom in each category indicated the median and 90th percentile of scores. **B** ROC curves of HOXL-score for ESCC and ESCA classification. The points indicated the best cutoff of HOXL-scores, and the percentages were the best specificity and sensitivity estimated by Youden’s index

We used logistic regression to develop an ESCC classification model based on HOXC10/HOXD1 methylation using the TCGA ESCC cohort. The risk score, defined as HOXL-score, was then estimated for each sample. The normal samples have the lowest risk scores than ESCC and ESCA (Fig. 5A, median: 0.28, 0.88, and 0.99). According to the mechanism of logistic regression, the HOXL score represents the probability that a sample is classified as cancer. Therefore, we attempted to classify ESCC and normal samples using HOXL scores. ROC curve analysis indicated that the AUC reached 0.96 (95% CI 0.91–0.99) for ESCC (Fig. 5B). The optimal threshold determined by the Youden index is 0.72, with a sensitivity of 94.8% and specificity of 87.5%. Besides, for ESCA, the AUC is 0.83 (95% CI 0.72–0.93), with sensitivity and specificity of 83.1% and 87.5% at the optimal threshold (Fig. 5B). No significant variations are observed for HOXL-score in detecting both ESCC and ESCA stratified by gender, age, and stage at the threshold of 0.72 (Table 3).

**Table 3:** The positive detection rates of HOXL-score for ESCC and ESCA with different clinical features

Features	Normal (%, total)	ESCC (%, total)	ESCA (%, total)	Χ^2^ *P*
Gender				1.00
Female	0.00% (n = 6)	93.33% (n = 15)	83.33% (n = 12)	
Male	20.00% (n = 10)	95.06% (n = 81)	83.12% (n = 77)	
Age				
< 50	100% (n = 1)	100% (n = 18)	83.33% (n = 6)	0.44
≥ 50	6.67% (n = 15)	93.59% (n = 78)	83.13% (n = 83)	
TNM stage				0.93
I–II		92.06% (n = 63)	85.29% (n = 34)	
III–IV		100% (n = 31)	79.41% (n = 34)	
Na		100% (n = 2)	85.71% (n = 21)	

The diagnostic performance of 13 potential methylation markers, *CDKN2A*, *CDKN2B*, *TFF1*, *MGMT*, *MLH1*, *DAPK1*, *SCGB3A1*, *TFPI2*, *DACH1*, *SOX17*, *CHFR*, *CDH1*, *APC* that have been reported in ESCC were also evaluated using the same approach in TCGA ESCC dataset (Additional file 1: Fig. S5). We observed the highest AUC value (0.96 [95% CI 0.91–0.98]) for *DAPK1* methylation, which is comparable to the HOXL-score. The AUC values of *CDKN2A* and *TFF1* are 0.85, which equals *HOXD1* but lower than *HOXC10*. These results suggest that the two genes and their combination show great potential for ESCC detection.

### Validation of the methylation status of *HOXD1* and *HOXC10* by sequencing

**Fig. 6:**
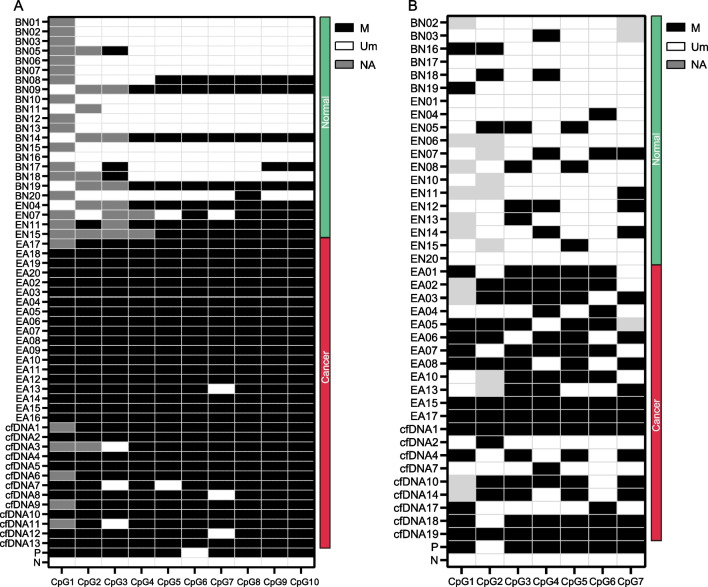
The methylation status of *HOXD1* (**A**) and *HOXC10* (**B**) between normal, ESCC-tissue and -plasma samples. Normal and ESCC samples were indicated by green and red side bars. BN, EN, EA, and cfDNA represented the blood samples, adjacent normal samples, ESCC-tissue and plasma samples, respectively. The methylated and unmethylated status of each CpG site were presented by black and white color in the heatmap. P and N indicated the positive and negative controls

Among all collected samples, amplification products of *HOXD1* were obtained from 4 normal samples, 19 ESCC tissues and 19 blood samples. For *HOXC10*, products were obtained from 13 normal samples, 12 cancerous tissues and 6 blood samples. The results of Sanger sequencing indicate that methylation events of the target regions on both genes occur more frequently on ESCC samples (Table 4). For all CpG sites, the methylation frequency on ESCC samples is significantly higher than that on normal samples (Additional file 1: Table S9). Sanger sequencing was also conducted for 13 cfDNA samples collected from ESCC patients’ plasma. In cfDNA samples, we observe that 90.77% (118/130) of *HOXD1* CpG sites are methylated, and 60.67% (37/61) of *HOXC10* CpG sites are methylated (Fig. 6A, B; Table 4). The methylation ratios of cfDNA samples are a little lower than tissue samples (99.47% for *HOXD1* and 73.42% for *HOXC10*), which might be attributed to the low cfDNA amount in plasma resulting in failed detection. Besides, we observe lower methylation levels of *HOXC10* than *HOXD1* on both tumor and normal samples in TCGA dataset, which is also revealed by the sequencing results that fewer methylation events occur on both tumor and normal samples for *HOXC10* than *HOXD1* (Table 4).

**Table 4:** The methylation frequency of the CpG sites in target regions

	Methylated CpGs	uMethylated CpGs	P value
*HOXD1*			
Normal	58	138	P < 0.001
ESCC-tissue	188	1	
ESCC-cfDNA	118	12	
*HOXC10*			
Normal	23	97	P < 0.001
ESCC-tissue	58	21	
ESCC-cfDNA	37	24

## Discussions

Abnormal DNA methylation is the most common epigenetic variation in human diseases and has been observed in various cancer types, including esophageal cancer. Therefore, further investigations of DNA methylation will facilitate understanding its role in tumor formation and developing more effective methylation-based biomarkers for cancer early detection. This study identified more than 40,000 differentially methylated CpG sites between ESCC and paired normal samples using WGBS data. A sliding window method was adopted to determine the differentially methylated regions and differentially methylated genes involved in various biological processes. We observe the frequent hypermethylation of HOXL subclass homeoboxes genes on ESCC, and the subsequent studies suggest their potential utility in discriminating ESCC from normal samples.

We identify 361,341 NC-DMCs and 69,278 ESCC-DMCs from the WGBS data covering 18 million CpGs, accounting for 2.44% of the total CpG sites, close to the previously reported 2.7% of methylated CpG sites [27]. Previous studies based on LINE-1, an alternative indicator of the whole genome, consistently observed genome-wide hypomethylation in ESCC [36]. Similar results are obtained in this study, with more NC-DMCs identified than ESCC-DMCs (approximately five times), suggesting the prevalent hypomethylation events in ESCC. The median distance between two adjacent ESCC-DMCs is smaller than that of two adjacent NC-DMCs, indicating that the ESCC-DMCs preferred to be more concentrated in one region. This feature is also evidenced by the fact that more ESCC-DMRs are identified (NC-DMRs/ESCC-DMRs: 6422/9040).

The more ESCC-DMRs identified than NC-DMRs can be explained by the algorithm parameters used in this study. We limit the maximum length of a DMR to 100 bp, based on the principle that the optimal amplicon length is around 100 bp when designing methylation-specific PCR primers. In a DMC-enriched region, a smaller maximum length threshold implies that more DMRs will be determined. For example, two DMRs (R1 and R2) are determined in a region of 114 bp in *HOXC10* gene when the maximum length is setting 100. However, when the maximum length is 300, only 1 DMR is determined (R1 and R2 are considered as one DMR). Therefore, it is reasonable to infer that more NC-DMRs will be identified when using a larger DMR maximum length. We then re-analyze the DMRs by setting the maximum length to 300, and as expected, more NC-DMRs are found (12099NC-DMRs vs 9008 ESCC-DMRs). Overall, we notice that the ESCC-DMRs are more closely distributed with each other and tend to aggregate to form a larger hypermethylated cluster.

The subsequently identified 733 NC-DMGs and 906 ESCC-DMGs include *PAX1* [37] and *STK3* [38], of which both have been attempted as diagnostic markers for ESCC. Functional enrichment results reveal that ESCC-DMGs are enriched in multiple biological processes, such as the reported cell cycle regulation and Wnt signaling pathways [36]. Notably, the top 3 enriched families, NKL subclass homeoboxes and pseudogenes, HOXL subclass homeoboxes and Zinc fingers, are frequently observed hypermethylated in ESCC. A couple of studies have demonstrated that the Zinc fingers family genes, such as *ZNF382*, *ZNF582*, *ZNF418,* and *ZNF542,* are methylated on ESCC [37–39]. However, the NKL and HOXL subclass, both of which belong to the homeoboxes superfamily, are not well reported. Our findings show the hypermethylation events of homeoboxes superfamily genes on ESCC, suggesting their potential roles in esophageal tumorigenesis.

This study reveals the widespread hypermethylation of HOXL subclass homeobox genes on ESCC in multiple independent datasets. Methylation of *HOXC10* and *HOXD1* show the best performance in discriminating ESCC from normal samples by ROC curve analysis. Although hypermethylation events of the two genes are rarely reported in ESCC by previous studies, they have been extensively studied in other cancer types, especially in breast cancer [40, 41]. Interestingly, in a recently published study, researchers have investigated the potential of *HOXC10* as a diagnostic marker for ESCC [42]. Using the WGBS technology, they identified the hypermethylated *HOXC10* in their cohort, which is consistent with the findings of this study.

Accumulating evidence demonstrated the crucial role of *HOXC10* in the development and progression of colorectal and gastric cancers [43, 44]. *HOXC10* was reported upregulated in ESCC, and its high expression contributed to the proliferation and migration of tumor cells, indicating that *HOXC10* could be an unfavorable prognostic predictor [45]. The current findings reveal the pervasive hypermethylation status of *HOXC10* on ESCC, which does not seem to support the silenced expression of *HOXC10* by epigenetics regulation. Growing evidence has demonstrated the complex relationship between gene methylation and expression. The methylated *CDKN2A* gene (also known as p16 locus), which encoded two genes, *p14ARF* and *p16INK4a*, has been attempted as a screening marker for ESCC [46]. Interestingly, its promoter hypermethylation only silenced the expression of *p14ARF* but not *p16INK4a* [20], implying that hypermethylation in different locations has various effects on gene expression. In general, the promoter hypermethylation events can downregulate gene expression, but it is not always the case. For example, the hypermethylated *SDC2* has been successfully commercialized for the early detection of colorectal cancer [47–49], while an apparent contradiction is that its expression increased in colorectal cancer, and the upregulated expression promotes cancer development [50–52]. Relatively, the relationship between hypermethylation events in gene body and expression is more complex. It has been reported that the hypermethylation events in homeobox gene bodies did not suppress expression but rather upregulated the expression to activate their oncogene activity [53]. In this study, DMRs of *HOXC10* are primarily located in the gene body, which may explain this phenomenon. In addition, miRNA and lncRNA may involve in regulating gene expression too. In gastric cancer, *HOXC10* was found to be a direct target of MiR-136 [54]. The downregulated MiR-136 led to the upregulation of *HOXC10*, thus increasing the risk of peritoneal metastasis. The antisense transcript, lncHOXC-AS3, was also reported associated with the regulation of *HOXC10* expression [55].

The hypermethylated *HOXD1* is rarely reported on ESCC in previous studies. However, in colorectal cancer, its hypermethylation is associated with the silenced expression and occurred along with the cancer formation [56]. The hypermethylation is also observed in breast cancer and used as a biomarker to detect this disease [40, 57]. In addition, methylated *HOXD1* is selected as a marker of lymph node metastasis in gastric cancer [58]. In this study, the Sanger sequencing results also reveal more frequently methylated events of *HOXD1* on ESCC samples than on normal samples. These findings suggest that *HOXD1* methylation can be a promising marker for ESCC detection.

We attempted to develop a classifier for ESCC by combing the methylation of *HOXL10* and *HOXD1*. The classifier obtains an AUC of 0.96 (95% CI 0.91–0.99), with a sensitivity of 94.8% and specificity of 87.5% at the optimal threshold of 0.72 determined by Youden index, suggesting its good performance in discriminating ESCC from normal samples. Furthermore, no significant variations are observed for the classifier in detecting ESCC with different age, gender, and pathological stages. However, the relationship between the classifier and patient features should be evaluated in a larger scale dataset as the results can be biased due to the small sample size. Notably, the classifier exhibits a lower detection rate for early-stage (I-II) than advanced stage ESCC (III-IV), which might be related to the higher methylation levels of *HOXL10* and *HOXD1* on advanced samples. Additionally, the classifier sensitivity for ESCA exceeds 80%, indicating its potential ability to detect ESCA.

Age is an important factor affecting DNA methylation. In the TCGA ESCC dataset (n = 94), we assessed the correlation of *HOXD1* and *HOXC10-*methylation with patient age. The *HOXD1* methylation shows a weak negative correlation with age (correlation coefficient =  − 0.19), but not significant (p = 0.056). While for *HOXC10*, no correlation is observed (correlation coefficient =  − 0.12, p = 0.25), and this is the same for the other three genes, *HOXC11*, *GSX1*, and *CDX21* (Additional file 1: Fig. S6). The healthy controls included in this study are younger than ESCC patients because the healthy blood donors tend to be younger people. However, no significant correlation between methylation and age suggests that patient age may have limited effects on the methylation of these five genes.

HOXL subclass genes belong to the superclass homeobox genes, which are characterized by sharing the homeobox sequence and play crucial roles in embryonic development and cell differentiation [59]. Many homeobox genes are found hypermethylated in different cancer types, including *HOXD1* and *HOXD10* [60, 61] that are also identified in this study. In a pan-cancer study of more than 4000 genomic profiles [53], approximately 43% of homeobox genes were reported strong correlations between the overexpression and the gene body hypermethylation, suggesting DNA hypermethylation may be an epigenetic regulator of their upregulated expressions. Since many malignancy cells are tightly associated with stem cells, this can partially explain the frequent methylation events of homeobox genes in multiple cancer types [59].

## Conclusions

Genome-wide methylation profiling allows us to interrogate the remodeling of DNA methylation during esophageal carcinogenesis from a landscape view. We observe the widespread hypomethylation events and frequent hypermethylation of HOXL subclass homeoboxes and Zinc finger family genes in ESCC. Two HOXL subclass homeoboxes, *HOXC10* and *HOXD1*, present good classification abilities for ESCC and normal samples. Early detection of ESCC is a challenging task. Many previous studies in epigenetics have paved a concrete road for unraveling the mechanism of ESCC carcinogenesis and identifying high-performed diagnostic markers. Our findings provide new insights to understand the epistatic remodeling and discover new methylation biomarkers for ESCC.

## Supplementary Information

**Additional file 1:** Supplementary figures and tables.

## Data Availability

The TCGA CRC 450 k data can be found in the Genomic Data Commons Data Portal (https://portal.gdc.cancer.gov/). GEO datasets can be found in the Gene Expression Omnibus (GEO, https://www.ncbi.nlm.nih.gov/geo/, GSE149608 and GSE52826) database. The BS-sequences of *HOXD1* and *HOXC10* were submitted to GenBank (Submission ID: 2585648 and 2631109). The other data generated in this study are fully reflected in the manuscript.

## Abbreviations

EC: Esophageal cancer
ESCA: Esophageal adenocarcinoma
ESCC: Esophageal squamous cell carcinoma
DMC: Differentially methylated CpGs
DMR: Differentially methylated regions
DMG: Differentially methylated genes
WGBS: Whole-genome bisulfite sequencing
TCGA: The Cancer Genome Atlas
MSP: Methylation specific PCR
